# Clinical implications of increased uptake in bone marrow and spleen on FDG-PET in patients with bacteremia

**DOI:** 10.1007/s00259-020-05071-8

**Published:** 2020-10-26

**Authors:** Jordy P. Pijl, Thomas C. Kwee, Riemer H. J. A. Slart, Derya Yakar, Marjan Wouthuyzen-Bakker, Andor W. J. M. Glaudemans

**Affiliations:** 1 Medical Imaging Center, Departments of Radiology, Nuclear Medicine and Molecular Imaging, University Medical Center Groningen, University of Groningen, Hanzeplein 1, P.O. Box 30.001, 9700 RB Groningen, The Netherlands; 2 TechMed Centre, Department of Biomedical Photonic Imaging, University of Twente, Enschede, The Netherlands; 3 Department of Medical Microbiology and Infection Prevention, University Medical Center Groningen, University of Groningen, Groningen, The Netherlands

**Keywords:** Bacteremia, Bone marrow, Spleen, FDG, Uptake, Sepsis

## Abstract

**Purpose:**

To investigate which clinical factors and laboratory values are associated with high FDG uptake in the bone marrow and spleen on 2-deoxy-2-[18F]fluoro-d-glucose (FDG) positron emission tomography (PET)/computed tomography (CT) in patients with bacteremia.

**Methods:**

One hundred forty-five consecutive retrospective patients with bacteremia who underwent FDG-PET/CT between 2010 and 2017 were included. Mean standard uptake values (SUV_mean_) of FDG in bone marrow, liver, and spleen were measured. Bone marrow-to-liver SUV ratios (BLR) and spleen-to-liver SUV ratios (SLR) were calculated. Linear regression analyses were performed to examine the association of BLR and SLR with age, gender, hemoglobin, leukocyte count, platelets, glucose level, C-reactive protein (CRP), microorganism, days of antibiotic treatment before FDG-PET/CT, infection focus, use of immunosuppressive drugs, duration of hospital stay (after FDG-PET/CT), ICU admission, and mortality.

**Results:**

C-reactive protein (*p* = 0.006), a cardiovascular or musculoskeletal focus of infection (*p* = 0.000 for both), and bacteremia caused by Gram-negative bacteria (*p* = 0.002) were independently and positively associated with BLR, while age (*p* = 0.000) and glucose level before FDG-PET/CT (*p* = 0.004) were independently and negatively associated with BLR.

For SLR, CRP (*p* = 0.001) and a cardiovascular focus of infection (*p* = 0.020) were independently and positively associated with SLR, while age (*p* = 0.002) and glucose level before FDG-PET/CT (*p* = 0.016) were independently and negatively associated with SLR.

**Conclusion:**

High FDG uptake in the bone marrow is associated with a higher inflammatory response and younger age in patients with bacteremia. In patients with high FDG uptake in the bone marrow, a cardiovascular or musculoskeletal focus of infection is more likely than other foci, and the infection is more often caused by Gram-negative species. High splenic FDG uptake is associated with a higher inflammatory response as well, and a cardiovascular focus of infection is also more likely in case of high splenic FDG uptake.

## Introduction

Infection is one of the most common reasons for hospital admission [1]. In patients with systemic signs of infection such as fever, blood cultures may be obtained to assess whether bacteria crossed anatomic barriers of the body and entered the bloodstream, causing bacteremia [2, 3]. With a 30-day mortality rate higher than 15%, bacteremia ranks among the top 10 causes of death in Europe and North America [4, 5].

2-Deoxy-2-[18F]fluoro-d-glucose (FDG) PET/CT can be used to evaluate the whole body for a focus of bacteremia in one examination. As white blood cells and other inflammatory cells are drawn to sites of infection and consume more glucose, infection sites are often readily visible on FDG-PET/CT, even before anatomical changes (such as abscess formation) have occurred [6]. In the last two decades, FDG-PET/CT has established its role in a broad spectrum of infectious diseases, including bacteremia (of unknown origin) [6, 7].

In some patients undergoing FDG-PET/CT for detecting the focus of infection, markedly increased FDG uptake is seen in the bone marrow or spleen. The bone marrow and spleen are generally regarded as “hypermetabolic” when their FDG avidity exceeds that of the liver (bone marrow/liver ratio or spleen/liver ratio > 1.0) [8]. Hypermetabolism of the bone marrow or spleen has been associated with laboratory values such as C-reactive protein or hemoglobin and even prognosis in patients with several types of cancer [9–14] and autoimmune disease [15, 16]. However, the clinical and pathophysiological meaning of hypermetabolism of the bone marrow and spleen in patients with bacteremia is limited. In a study by Ahn et al., FDG uptake in the spleen seemed a useful measure to discriminate between autoimmune disease and bacteremia [16]. And, in a study by Boursier et al., high FDG uptake of the bone marrow and spleen was independently associated with endocarditis [17].

In current clinical practice, high FDG uptake of the bone marrow or spleen is generally not taken into account in patients with bacteremia undergoing FDG-PET/CT for finding the focus of infection because the implications of this finding remain largely unknown in this patient category.

In this study, we investigated the pathophysiological and clinical implications of high FDG uptake in the bone marrow and spleen on FDG-PET/CT in a large number of patients with bacteremia.

## Materials and methods

### Study design and patients

The electronic patient database of the University Medical Center Groningen was searched for all patients with bacteremia who underwent FDG-PET/CT for finding the focus of infection between 2010 and 2017 using the keywords “sepsis,” “bacteremia,” “infection focus,” and “blood culture.” Patients were included if they had positive blood cultures within 2 months before FDG-PET/CT was performed. Patients were excluded if they had positive blood cultures that were interpreted as contamination by the medical microbiologist, if FDG-PET images were not available for quantitative measurements, if patients had malignant disease such as leukemia or lymphoma (as these patients may have abnormal FDG uptake in the bone marrow or spleen due to their cancer), and if they had glucose levels above 11 mmol/L (198 mg/dL) before FDG administration. Follow-up FDG-PET/CT scans and FDG-PET/CT scans performed for other reasons than locating the source of infection (such as oncologic follow-up) were excluded as well.

The Medical Ethics Review Board of the University Medical Center Groningen approved this retrospective, single-center study and waived the requirement for written informed consent (Institutional Review Board number 201700145). The patient cohort analyzed in this study is previously described [18].

### Patient data review

The medical files of all patients were reviewed for relevant clinical and biochemical data including age, gender, medical history, laboratory values (hemoglobin, platelets, CRP, leukocyte count, glucose level, type of bacteria), imaging results and procedures, admission to intensive care unit, medication and treatment, and follow-up data. The final diagnosis was based on all available clinical data and diagnostic test results including histology and microbiology reports, FDG-PET/CT results and other types of imaging that were performed, clinical response to treatment, and follow-up for at least 6 months.

### FDG-PET/CT acquisition

All scans were performed using an integrated PET/CT system (Biograph mCT 40 or 64 slice PET/CT; Siemens, Knoxville, TN, USA) with 3 min per bed position according to European Association of Nuclear Medicine guidelines [19]. Patients fasted for a minimum of 6 h before 3 MBq FDG/kg body weight was administered intravenously. When there was a clinical suspicion of infective endocarditis, patients were also prepared with a high-fat, low-carbohydrate diet for at least 24 h. PET/CT imaging was performed approximately 60 min after intravenous FDG administration.

Low-dose CT was performed for attenuation correction and anatomic mapping with 100 kV and 30 mAs. In 28 patients, concomitant full-dose CT of neck, thorax, or abdomen was performed with a constant tube potential of 100 or 120 kV and automatic adjustment of mAs in the z-direction.

### Quantitative FDG-PET measurements

In all included patients, mean standardized FDG uptake values (SUV_mean_) were determined using Syngovia software (Siemens Healthcare, Erlangen, Germany). Bone marrow SUV_mean_ values were measured with spherical volumes of interest (VOI) at four anatomical landmarks (the right femur head, the corpus of the first thoracic and first lumbar vertebrae, and the right posterior iliac crest) and then averaged. The right-sided femur head and posterior iliac crest were chosen as most patients were presumed to be right limb dominant. The VOI diameters were set in the bone marrow, excluding cortical bone and entities on CT such as Schmorl’s nodes in the vertebrae and bone islands. Splenic and hepatic FDG uptake was measured using VOIs at the center of the spleen and center of the right liver lobe, excluding large vessels and lesions such as cysts and hemangiomas. The liver was used as a reference organ for FDG uptake in the bone marrow and spleen. Thus, bone marrow-to-liver SUV ratios (BLR) and spleen-to-liver SUV ratios (SLR) were calculated. All SUVs were corrected for plasma glucose levels according to the European Association of Nuclear Medicine guidelines and all scans were reconstructed according to resEARch 4 Life [19].

### Statistical analysis

For baseline data, all continuous variables were checked for normal distribution using Kolmogorov-Smirnov tests. Data were presented as mean ± standard deviation or median with interquartile range (IQR) for normally distributed or non-normally distributed data, respectively.

To investigate which factors were associated with bone marrow and splenic FDG uptake, univariate linear regression and multivariate (stepwise) linear regression analyses were performed. BLR and SLR were selected as the dependent variable. Age, gender, hemoglobin, leukocyte count, platelets, glucose level, CRP, type of bacteria, days of antibiotic treatment, infection focus, use of immunosuppressive drugs, duration of hospital stay (after FDG-PET/CT), ICU admission, and mortality were used as independent variables. *P* values of < 0.05 were considered statistically significant.

All statistical analyses were performed using IBM Statistical Package for the Social Sciences (SPSS) version 25 (SPSS, Chicago, IL).

## Results

### Patient population

**Fig. 1:**
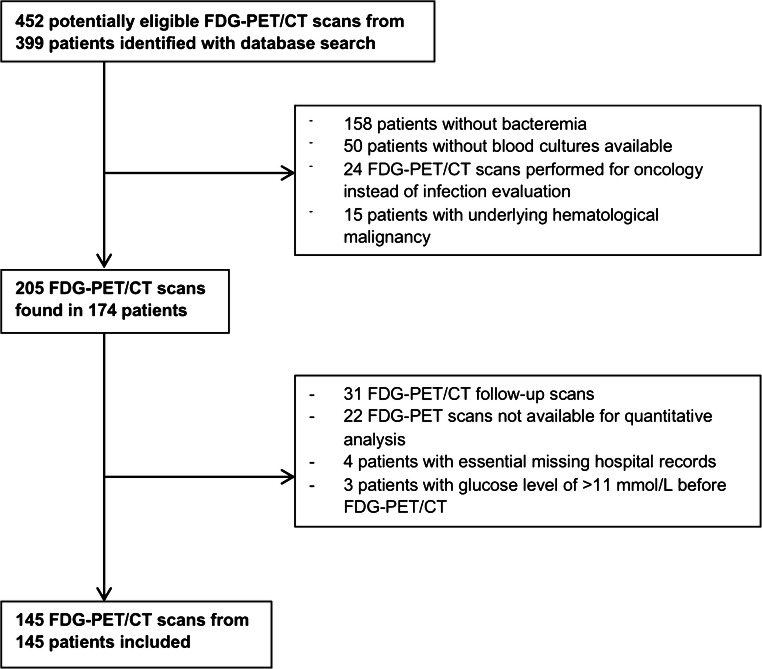
After review of the inclusion and exclusion criteria, 145 FDG-PET/CT scans from 145 patients were finally included

Four hundred fifty-two FDG-PET/CT scans from 399 individual patients were potentially eligible for inclusion. After review of the inclusion and exclusion criteria, 145 FDG-PET/CT scans from 145 patients were finally included (Fig. 1).

**Fig. 2:**
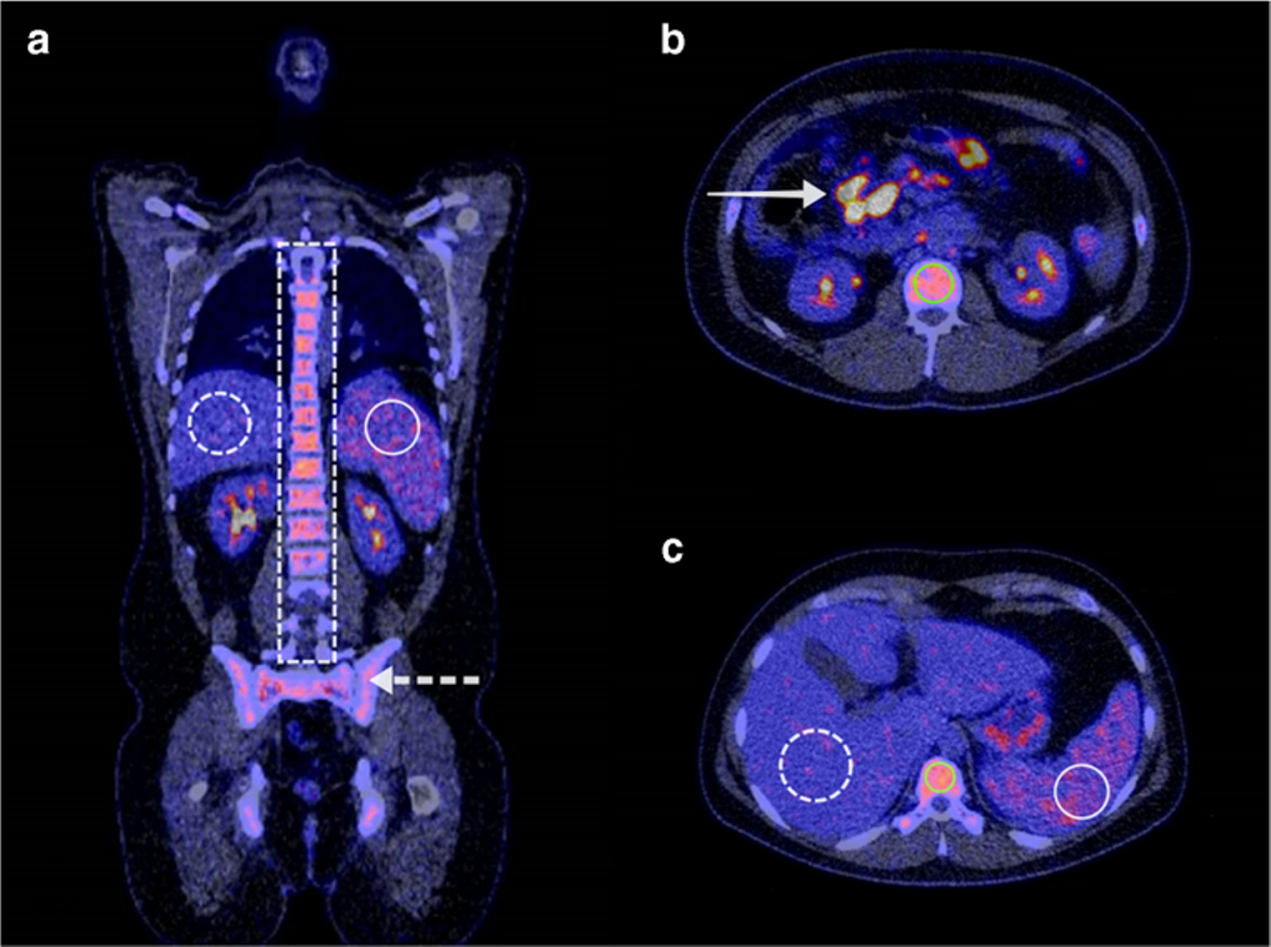
A 26-year-old man with no previous medical history presented at the hospital with a fever of 39 °C and watery diarrhea after a holiday to South America. A complete physical exam and a thoracic X-ray showed no abnormalities. His leukocyte count was 3.8 × 10^9^/L, and his CRP level was 165 mg/L. Blood cultures were positive for *Salmonella* species. Antibiotic treatment was initiated with ceftriaxone. Because his clinical condition did not improve, FDG-PET/CT was performed to identify the focus of infection. FDG-PET/CT showed diffuse high FDG uptake in the bone marrow, including the spinal vertebrae (A, dashed white rectangle; B, C, green circle) and pelvis (A, dashed white arrow) with a BLR of 1.40. FDG uptake in the spleen (A, C, white circle) was also higher than in the liver (A, C, dashed white circle) with an SLR of 1.26. Apart from mesenteric lymphadenitis (B, white arrow), no other infection focus was found. Antibiotic therapy was continued, and the patient was discharged 1 week after FDG-PET/CT in good clinical condition

**Fig. 3:**
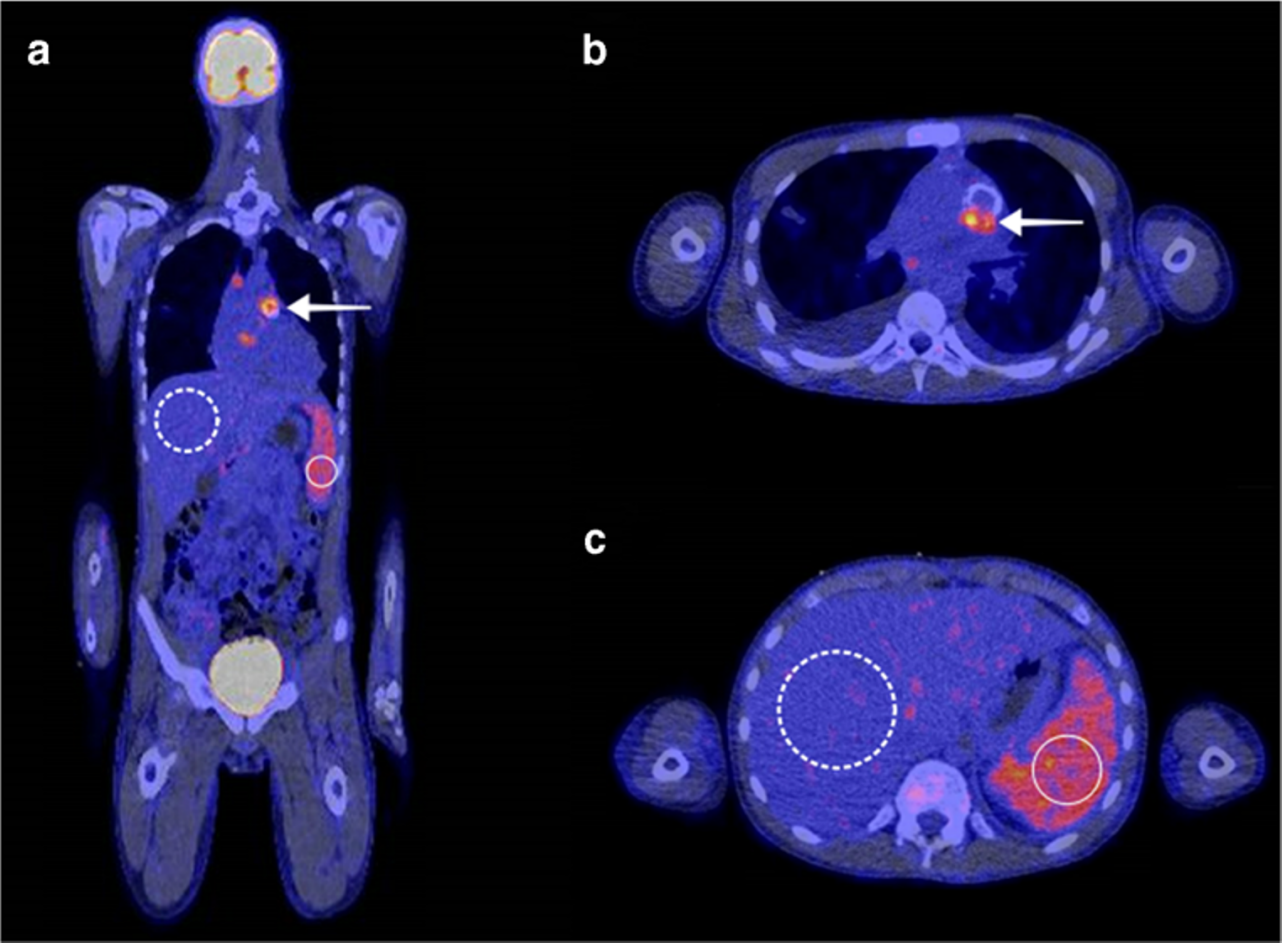
A 14-year-old boy presented to the hospital with an intermittent fever up to 40 °C, fatigue, and painful muscles in his legs for approximately 2 weeks. His medical history included aortic valve replacement with an artificial valve. On auscultation, a systolic murmur was heard, which was loudest at the third intercostal space on the left side of the sternum. His leukocyte count was 13.2 × 10^9^/L, and his CRP level was 40 mg/L. Blood cultures were positive for *Streptococcus parasanguinis*. A possible aortic valve vegetation was seen on a transthoracic ultrasound. On FDG-PET/CT, pathologic FDG uptake of the aortic valve was seen (A, B, white arrow), confirming the diagnosis of artificial valve endocarditis. FDG uptake in the spleen (white circle) was evidently higher than in the liver (dashed white circle), with an SLR of 1.73 and a BLR of 1.11. Despite adequate antibiotic treatment, there was no clinical improvement, and the aortic valve was surgically replaced 2 weeks after FDG-PET/CT. Six weeks after the surgery, the patient could be discharged from the hospital in good clinical condition

These included 90 men and 55 women, with a median age of 64 years (IQR 22), median CRP level of 84 mg/L (IQR 100), and median leukocyte count of 10.0 × 10^9^/L (IQR 6.6). In all patients, bacteremia was most commonly caused by *Staphylococcus aureus* (40 patients, 28% of total), Gram-negative species (34 patients, 23% of total), and coagulase-negative staphylococci (19 patients, 13% of total) (Table 1**)**. The most common foci of infection were musculoskeletal (31 patients, 21% of total), cardiovascular (28 patients, 19% of total), and hepatopancreaticobiliary (20 patients, 14% of total) (Table 2). Forty patients (28% of total) used immunosuppressive drugs at the time of the FDG-PET/CT, and 139 patients (96%) received antibiotic treatment before FDG-PET/CT. The median total duration of hospital stay was 23 days (IQR 27), and patients remained in the hospital for a median of 13 days (IQR 18) after FDG-PET/CT. Twenty-two patients (15% of total) were admitted to the ICU during hospitalization, and 19 patients (13% of total) died during hospital admission. The FDG-PET/CT images of two exemplary patients are shown in Figs. 2 and 3.

**Table 1:** Baseline characteristics

Characteristic	Value (*n* = 145)
Age (years)	64.0 (22)^a^
Gender	
Men	90 (62%)
Women	55 (38%)
Hemoglobin (mmol/L)	6.2 (1.5)^a^
C-reactive protein (mg/L)	84.0 (100)^a^
Leukocytes (× 10^9^/L)	10.0 (6.6)^a^
Glucose level (mmol/L)	5.2 (1.5)^a^
Isolated bacteria from blood culture	
*Staphylococcus aureus*	40 (28%)
Gram negatives	34 (23%)
Coagulase-negative staphylococci	19 (13%)
*Enterococcus* species	18 (12%)
*Streptococcus* species	15 (10%)
Other	2 (1%)
Polymicrobial	17 (12%)
Patients admitted to ICU	22 (15%)
Patients using immunosuppressive medication	40 (28%)
Patients receiving antibiotic treatment before FDG-PET/CT	139 (96%)
Days of antibiotic treatment before FDG-PET/CT	7 (9)^a^
Days between FDG-PET/CT and hospital discharge	13 (18)^a^
Total hospital stay (days)	23 (27)^a^
Infection focus based on final diagnosis	
Musculoskeletal	31 (21%)
Cardiovascular	28 (19%)
Hepatopancreaticobiliary	20 (14%)
Gastrointestinal	9 (6%)
Catheter/drain	8 (6%)
Pulmonary	8 (6%)
Urogenital	7 (5%)
Unknown	34 (23%)
In-hospital mortality	19 (13%)

^a^Median (interquartile range)

**Table 2:** Final diagnosis at hospital discharge

Type of infection	Number of patients
Musculoskeletal	31 (21%)
Spondylodiscitis	21 (14%)
Sacroiliitis	3 (2%)
Septic arthritis	3 (2%)
Sternoclavicular infection	2 (1%)
Mediastinitis	1 (1%)
Psoas abscess	1 (1%)
Cardiovascular	28 (19%)
Endocarditis	15 (10%)
Vascular graft infection	7 (5%)
Septic thrombophlebitis	2 (2%)
Mycotic aneurysm	2 (2%)
Pericarditis	1 (1%)
Aortitis	1 (1%)
Hepatopancreaticobiliary	20 (14%)
Infected liver cyst	6 (4%)
Cholangitis	5 (3%)
Cholecystitis	3 (2%)
Liver abscess	3 (2%)
Necrotizing pancreatitis	2 (2%)
Surgical site infection after hemihepatectomy	1 (1%)
Gastrointestinal	9 (6%)
Esophagitis	2 (2%)
Mesenteric lymphadenitis	1 (1%)
Enterocolitis	1 (1%)
Diverticulitis	1 (1%)
Pseudomembranous colitis	1 (1%)
Sigmoiditis	1 (1%)
Ileum perforation	1 (1%)
Catheter/drain/other	8 (6%)
Infected intravenous catheter	5 (3%)
Infected venous access point	1 (1%)
Infected nephrostomy catheter	1 (1%)
Infected gamma nail	1 (1%)
Pulmonary	8 (6%)
Pneumonia	6 (4%)
Aspergilloma	1 (1%)
Q fever	1 (1%)
Urogenital	7 (5%)
Pyelonephritis	3 (2%)
Infected kidney cyst	2 (2%)
Kidney abscess	2 (2%)
Unknown origin	34 (23%)

### Quantitative analysis FDG uptake

The median SUV_mean_ of FDG in the bone marrow was 1.70 (IQR 0.81) among all included patients. The median SUV_mean_ in the spleen was 2.44 (IQR 0.99) and 2.29 (IQR 1.07) in the right liver lobe. The median BLR was 0.76 (IQR 0.40), and 38 patients (26%) showed increased FDG uptake in the bone marrow (BLR > 1.00). The median SLR was 1.01 (IQR 0.31), with 75 patients (52%) showing increased FDG uptake in the spleen (SLR > 1.00). In 32 patients (22%), both BLR and SLR were elevated with ratios higher than 1.00 (Table 3).

**Table 3:** Quantitative analysis of FDG uptake

Parameter	Value
Bone marrow SUV_mean_^a^	1.70 (0.81)^b^
Spleen SUV_mean_	2.44 (0.99)^b^
Right liver lobe SUV_mean_	2.29 (1.07)^b^
Bone marrow-to-liver ratio	0.76 (0.40)^b^
Patients with bone marrow-to-liver ratio > 1.00	38
Range of bone marrow-to-liver ratio	
Lowest	0.37
Highest	2.22
Spleen-to-liver ratio	1.01 (0.31)^b^
Patients with spleen-to-liver ratio > 1.00	75
Range of spleen-to-liver ratio	
Lowest	0.64
Highest	2.45
Patients with bone marrow-to-liver ratio and spleen-to-liver ratio > 1.00	32

^a^Averaged SUV_mean_ of the right humeral head, first thoracic vertebra, first lumbar vertebra, and right posterior iliac crest

^b^Median (interquartile range)

### Variables associated with increased bone marrow FDG uptake

On univariate linear regression, BLR was significantly associated with 14 individual variables.

Duration of hospital stay after FDG-PET/CT, ICU admission, bacteremia caused by Gram-negative or multiple bacteria, leukocyte count, CRP, platelets, detecting any focus of infection on FDG-PET/CT, and having a cardiovascular or musculoskeletal focus of infection were positively associated with BLR. Age, duration of antibiotic treatment before FDG-PET/CT, use of immunosuppressive drugs, and glucose level were negatively associated with BLR (Table 4). To identify which factors had the strongest independent association with high bone marrow uptake, multivariate (stepwise) linear regression analysis was performed.

**Table 4:** Univariate linear regression between bone marrow-to-liver ratio (BLR), spleen-to-liver ratio (SLR), and clinical (infection) parameters

Variable	BLR			SLR		
	Unstandardized B	95% CI	Sig.	Unstandardized B	95% CI	Sig.
Age (years)	− 0.007	− 0.010 to − 0.005	0.000	− 0.003	− 0.006 to − 0.001	0.002
Male gender	− 0.015	− 0.12 to 0.093	0.786	0.004	− 0.087 to 0.095	0.93
Duration of stay after PET (days)	0.004	0.001 to 0.007	0.015	0.002	0.000 to 0.005	0.068
Days of antibiotic treatment	− 0.008	− 0.015 to − 0.001	0.026	− 0.003	− 0.009 to 0.003	0.26
Using immunosuppressive drugs	− 0.12	− 0.24 to − 0.008	0.036	− 0.125	− 0.22 to − 0.030	0.010
ICU admission	0.25	0.101 to 0.39	0.001	0.12	− 0.001 to 0.24	0.051
Mortality	− 0.12	− 0.274 to 0.032	0.120	− 0.083	− 0.21 to 0.048	0.21
Microorganism						
Coagulase negative	Reference category			Reference category		
*Enterococcus* species	− 0.021	− 0.22 to 0.18	0.83	0.062	− 0.11 to 0.23	0.48
Gram negatives	0.23	0.054 to 0.40	0.011	0.19	0.044 to 0.34	0.012
*S. aureus*	0.14	− 0.027 to 0.31	0.098	0.15	0.004 to 0.30	0.044
*Streptococcus* species	0.16	− 0.054 to 0.37	0.14	0.16	− 0.020 to 0.34	0.081
Polymicrobial	0.23	0.021 to 0.43	0.031	0.18	0.001 to 0.35	0.049
Other	− 0.028	− 0.48 to 0.43	0.90	0.16	− 0.020 to 0.34	0.081
Laboratory values						
Hemoglobin (mmol/L)	− 0.007	− 0.052 to 0.038	0.75	− 0.005	− 0.043 to 0.033	0.80
Leukocyte count (× 10^9^/L)	0.012	0.005 to 0.019	0.001	0.008	0.002 to 0.014	0.009
CRP (mg/L)	0.001	0.000 to 0.002	0.001	0.001	0.000 to 0.001	0.001
Platelets (× 10^9^/L)	0.000	0.000 to 0.001	0.038	0.000	0.000 to 0.000	0.23
Glucose level (mmol/L)	− 0.071	− 0.11 to − 0.032	0.001	− 0.051	− 0.085 to − 0.017	0.003
Detection of any focus	0.20	0.098 to 0.30	0.000	0.12	0.030 to 0.21	0.009
Focus of infection						
Unknown origin	Reference category			Reference category		
Gastrointestinal	0.13	− 0.083 to 0.35	0.23	0.066	− 0.12 to 0.26	0.49
Pulmonary	0.18	− 0.047 to 0.40	0.12	0.14	− 0.057 to 0.34	0.16
Cardiovascular	0.32	0.18 to 0.47	0.000	0.17	0.047 to 0.30	0.007
Musculoskeletal	0.25	0.11 to 0.39	0.001	0.14	0.022 to 0.27	0.021
Hepatopancreaticobiliary	0.13	− 0.031 to 0.29	0.11	− 0.026	− 0.17 to 0.11	0.71
Central line/catheter	− 0.046	− 0.27 to 0.18	0.69	− 0.10	− 0.30 to 0.096	0.31

For BLR, age showed the strongest independent association (0.007 decrease in ratio per year increase), followed by CRP (0.001 increase per unit increase in CRP), having a cardiovascular (0.25 increase) or musculoskeletal (0.20 increase) focus of infection, suffering from bacteremia caused by Gram-negative bacteria (0.15 increase), and glucose level before FDG-PET/CT (0.045 decrease in ratio per mmol/L increase). Together, these variables had an adjusted *R*^2^ of 0.46 (Table 5). No clinical outcome variables were independently associated with BLR.

**Table 5:** Multivariate stepwise linear regression between bone marrow-to-liver ratio (BLR) and clinical (infection) parameters

Step in regression	Variable	*R*^a^	Adjusted *R*^2a^	Unstandardized B (95% CI)	*β* coefficient	Sig.
1	Age (years)	0.47	0.22	− 0.007 (− 0.009 to − 0.005)	− 0.446	0.000
2	CRP (mg/L)	0.55	0.30	0.001 (0.000–0.001)	0.18	0.006
3	Cardiovascular focus	0.61	0.36	0.25 (0.15 to 0.36)	0.34	0.000
4	Musculoskeletal focus	0.64	0.40	0.20 (0.09 to 0.305)	0.273	0.000
5	Gram-negative bacteria	0.67	0.43	0.15 (0.054 to 0.246)	0.195	0.002
6	Glucose before PET (mmol/L)	0.69	0.46	− 0.045 (− 0.076 to − 0.014)	− 0.174	0.004

^a^In each subsequent step of the regression (steps 1 to 6), another variable is added to the model. The *R* and adjusted *R*^2^ show the cumulative effect of the included variables at each step. In step 6, all variables from the table are included

### Variables associated with increased spleen FDG uptake

On univariate linear regression, SLR was significantly associated with 11 individual variables. Bacteremia caused by Gram-negative bacteria, *Staphylococcus aureus*, or multiple bacteria, leukocyte count, CRP, detecting any focus of infection on FDG-PET/CT, and having a cardiovascular or musculoskeletal focus of infection were positively associated with SLR. Age, use of immunosuppressive drugs, and glucose level were negatively associated with BLR (Table 4). On multivariate (stepwise) linear regression, CRP showed the strongest independent association with SLR (0.001 increase in ratio per unit increase in CRP), followed by age (0.003 decrease in ratio per year increase), glucose level before PET (0.039 decrease per unit increase in glucose), and having a cardiovascular focus of infection (0.12 increase). Together, these variables had an adjusted *R*^2^ of 0.19. No clinical outcome variables were independently associated with SLR (Table 6).

**Table 6:** Multiple stepwise linear regression between spleen-to-liver ratio (SLR) and (clinical) infection parameters

Step in regression	Variable	*R*^a^	Adjusted *R*^2a^	Unstandardized B (95% CI)	*β* coefficient	Sig.
1	CRP (g/L)	0.27	0.065	0.001 (0.000 to 0.001)	0.249	0.001
2	Age (years)	0.38	0.13	− 0.003 (− 0.005 to − 0.001)	− 0.239	0.002
3	Glucose before PET (mmol/L)	0.42	0.16	− 0.039 (− 0.071 to − 0.007)	− 0.186	0.016
4	Cardiovascular focus	0.46	0.19	0.12 (0.019 to 0.22)	0.177	0.020

^a^In each subsequent step of the regression (steps 1 to 4), another variable is added to the model. The *R* and adjusted *R*^2^ show the cumulative effect of the included variables at each step. In step 4, all variables from the table are included

## Discussion

Our study included 145 patients with confirmed bacteremia who underwent FDG-PET/CT to find the focus of infection. In 38 patients (26%), FDG uptake was increased in the bone marrow (BLR > 1.00), and in 75 patients (52%), FDG uptake was increased in the spleen (SLR > 1.00). In 32 patients (22%), FDG uptake was increased in both the bone marrow and spleen. With multivariate linear regression, age was the strongest factor independently associated with BLR, followed by CRP, having a cardiovascular of musculoskeletal focus of infection, having bacteremia caused by Gram-negative bacteria, and blood glucose level before FDG-PET/CT. For SLR, CRP was the strongest independently associated factor, followed by age, blood glucose level before FDG-PET/CT, and having a cardiovascular focus of infection.

From a pathophysiological perspective, young age and high CRP appear to be the most important explanatory factors for a high BLR or SLR. It is known that bone marrow composition changes with aging. “Red bone marrow,” consisting of 60% hematopoietic cells and 40% fat cells, gradually progresses with age to “yellow bone marrow” which consists of 95% fat cells and 5% nonfat cells [20]. Because hematopoietic cells have a higher glucose metabolism than fat cells, more FDG accumulates in the bone marrow of younger people undergoing FDG-PET/CT, also when they are healthy. In patients with infectious disease, myelopoiesis and especially granulopoiesis are stimulated more than in healthy patients, leading to increased production of monocytes and granulocytes [21]. This process seems to amplify the effect of young age on the bone marrow-to-liver ratio in patients with bacteremia. Surprisingly, the hemoglobin level was not significantly associated with BLR or SLR, while high FDG uptake in the bone marrow or spleen has been associated with anemia in patients with cancer [22–24]. Erythropoiesis is often suppressed during infection [25], which may be the reason why a high BLR or SLR is not associated with hemoglobin level in patients with bacteremia. However, the etiology of the anemia should also be taken into account. For example, if patients with bacteremia were already anemic due to chronic disease, erythropoiesis may have already been suppressed before their episode of bacteremia [26].

Higher CRP levels were also independently associated with a high BLR and SLR. This finding supports the hypothesis that bone marrow and splenic FDG uptake are indeed related to the severity of infection, or at least a heavier inflammatory response to infection. Having bacteremia caused by Gram-negative bacteria also resulted in a higher BLR than other microorganisms. This may be explained by previous literature which states that bacteremia with Gram-negative bacteria elicits a more severe immune response than Gram-positive bacteria [27]. The spleen is especially involved in the clearance of encapsulated bacteria [28]. These can be both Gram-positive or Gram negative, which may be why bacteremia with Gram-negative bacteria did not cause a higher SLR than bacteremia with other encapsulated bacteria, such as *Streptococcus pneumoniae* or *Staphylococcus aureus*.

From a clinical perspective, it is an important finding that a cardiovascular focus of infection was independently associated with a higher BLR and SLR. The most common cardiovascular infections in our study population were endocarditis, vascular graft infection, septic thrombophlebitis, and mycotic aneurysm. A possible explanation may be that these infections may elicit a higher immune response than other infections. With cardiovascular infections such as endocarditis and infected vascular grafts, bacteria are constantly hematogenously spread through the body. Eradication of the pathogen usually requires up to 6 weeks of intravenous antibiotic therapy [29]. These infections are usually considered severe and as such may also evoke a more severe inflammatory response throughout the body, including the bone marrow and spleen. Therefore, physicians should be especially attentive to a potential cardiovascular focus of infection in case of a high BLR or SLR. Additionally, a musculoskeletal infection was also associated with a high BLR but not a high SLR. The reason for this observation remains unclear. However, the majority of musculoskeletal infections in this study consisted of spondylodiscitis. Although infected vertebrae were excluded from SUV measurements, it can be speculated that spondylodiscitis may also cause higher FDG uptake in other (adjacent) vertebral bone marrow due to the accumulation of inflammatory cells. This requires further investigation.

Lastly, higher glucose levels before FDG-PET/CT were also independently associated with a lower BLR and SLR. The liver plays a key role in glucose regulation [30]. In patients with higher glucose levels, glycogenesis is stimulated, causing more FDG to be phosphorylated to FDG-6-phosphate and be “trapped” in the liver to lower blood glucose levels [31]. This causes a higher increase in FDG uptake in the liver than in the bone marrow and spleen and therefore lower bone marrow-to-liver and spleen-to-liver ratios. Treatment with corticosteroids can also increase glucose level and hepatic FDG uptake [29], but BLR or SLR was not independently associated with receiving immunosuppressive drugs (of which 85% were corticosteroids) in our patient population.

Previous literature about the relation between BLR, SLR, and clinical or pathophysiological factors is very limited in patients with infectious disease. In a study by Inoue et al. [8], high BLR was also significantly associated with high CRP and young age in 65 patients, but most of these patients did not suffer from infectious disease. In a study by Ahn et al. [16], FDG uptake in the spleen was higher in 38 patients with macrophage activation syndrome than in 15 patients with sepsis. However, the patients with macrophage activation syndrome were significantly younger than the patients with sepsis, which may have influenced these results. In another study by Ahn et al. [15], high BLR and SLR were correlated with high CRP in 101 patients with localized infection, and BLR and SLR were higher in 91 patients with the autoimmune disease than in the patients with localized infection. Again, the patients with autoimmune disease were significantly younger than the patients with localized infectious disease, which may have influenced the results, especially since age showed a strong association with BLR and SLR in our study population.

In a recent study by Boursier et al. which included 129 patients with suspected endocarditis, high FDG uptake of the bone marrow and spleen was independently associated with “definitive” infective endocarditis [17]. Obesity and high blood glucose levels resulted in a lower BLR or SLR and having bacteremia resulted in a higher BLR or SLR. Because bone marrow and spleen FDG uptake were grouped together, the relation between the included variables and FDG uptake of the bone marrow or spleen individually is unclear. Also, 30% of the included patients did not have bacteremia, which makes the results difficult to translate to all patients with bacteremia.

In several types of cancer, increased FDG avidity of the bone marrow seemed to be associated with prognostic factors such as lower overall survival rate and higher cancer recurrence rate [9–14]. In our cohort of patients with bacteremia, BLR or SLR was not associated with a total duration of hospital stay, ICU admission, or mortality. There is no previous literature about the relation between BLR or SLR and clinical outcome in patients with infectious disease.

This study was compromised by some limitations. First, due to its retrospective design, there may have been selection bias. All included patients had bacteremia of unknown origin, and they underwent FDG-PET/CT to locate the source of infection. Patients with bacteremia who already have an established focus of infection may not undergo FDG-PET/CT. Therefore, it is uncertain whether the results of this study also apply to patients with bacteremia of known origin. Second, patients who were suspected of endocarditis were instructed to limit carbohydrate intake for 24 h and fast for 6 h to suppress myocardial FDG uptake, but some patients still had high glucose levels before FDG-PET/CT. Therefore, it seems likely that some patients did not follow the dietary instructions, which could have affected FDG-PET/CT results. Lastly, the human inflammatory response to bacteremia is a very complex process involving many different inflammatory mediators. Although this study did include the most important clinical factors (such as CRP and leukocytes), some specific laboratory markers were not determined in most patients due to the retrospective nature of this study and, therefore, not analyzed. With multivariate linear regression, BLR reached an adjusted *R*^2^ of 0.46, indicating that 46% of the variance in BLR can be explained by age, CRP, focus of infection, type of bacteria, and glucose level before FDG-PET/CT. For SLR, age, CRP, focus of infection, and glucose before FDG-PET/CT reached an adjusted *R*^2^ of 0.19. Because only 19% of SLR could be explained by our included factors, it is likely that other inflammatory markers (such as growth factors and cytokines) also play an important explanatory role in FDG uptake of the spleen in patients with bacteremia [32, 33]. It is known that cytokines play a role in upregulation of active glucose transporters (GLUT) in the plasma membrane of cells, which also increases FDG uptake of these cells [34]. However, the association between specific cytokines and FDG uptake in bone marrow or the spleen is not described in the current literature and cannot be explored by our study either due to its retrospective nature. Future prospective studies should include additional variables such as specific cytokines, growth factors, and progenitor cells involved in hematopoiesis to further enhance our understanding of high FDG uptake in the bone marrow and spleen of patients with bacteremia.

## Conclusion

High FDG uptake in the bone marrow is associated with a higher inflammatory response and younger age in patients with bacteremia. In patients with high FDG uptake in the bone marrow, a cardiovascular or musculoskeletal focus of infection is more likely than other foci, and the infection is more often caused by Gram-negative species. High splenic FDG uptake is associated with a higher inflammatory response as well, and a cardiovascular focus of infection is also more likely in case of high splenic FDG uptake.

### Availability of data and material

Due to patient confidentiality, the database will not be made available online. The corresponding author can be contacted to discuss possibilities on sharing data included in this study.
